# Improve the roles of nature reserves in conservation of endangered pheasant in a highly urbanized region

**DOI:** 10.1038/s41598-020-74724-3

**Published:** 2020-10-19

**Authors:** Kai Song, Chun-Rong Mi, Nan Yang, Lei Sun, Yue-Hua Sun, Ji-Liang Xu

**Affiliations:** 1 School of Ecology and Nature Conservation, Beijing Forestry University, Beijing, 100083 China; 2 Key Laboratory of Animal Ecology and Conservation Biology, Institute of Zoology, Chinese Academy of Science, Beijing, 100101 China; 3 Department of Ecology and Genetics, Uppsala University, Norbyvägen 18D, 75236 Uppsala, Sweden; 4 Baihuashan National Nature Reserve, Beijing, 102300 China; 5 Xiaowutaishan National Nature Reserve, Hebei, 075061 China

**Keywords:** Ecology, Conservation biology

## Abstract

Nature reserves play an extraordinarily important role in conserving animal populations and their habitats. However, landscape change and unreasonable zoning designations often render these protected areas inadequate. Therefore, regular evaluation of the efficacy of protected lands is critical for maintaining and improving management strategies. Using species distribution models and GAP analysis, we assessed the changes in suitable habitat for the Brown Eared-pheasant (*Crossoptilon mantchuricum*) in two Chinese nature reserves between 1995 and 2013. Our results showed that the habitat suitability of Brown Eared-pheasant has changed dramatically during this period, and fragmentation analyses showed an increase in concentration area and decrease in patch area. In particular, our findings show that the national nature reserves need to adjust their ranges to ensure the conservation of this flagship species. Our study further provides a new viewpoint for evaluating the efficacy of protected lands, particularly in highly urbanized regions where conservation goals must be balanced with changing landscapes.

## Introduction

Rapid economic development and human population growth over the last century have greatly intensified the threats to Earth’s ecosystems, and especially to endangered species^1,2^. Finding a balance between conservation and development is still a challenge globally, and even more difficult in countries with large populations such as China^3^. The Jing-Jin-Ji (JJJ) region is the national capital region of China, and is the biggest urbanized region in Northern China, which includes an economic region surrounding Beijing, Tianjin, and Hebei. In 2016, JJJ region produced 10% of China’s GDP, which is in part due to recent, extensive infrastructure projects^4,5^. As a recently emerged economic center, the JJJ region has seen expanded land use for agriculture, logging, and human settlement throughout a group of developed and urbanized cities. Land use changes and associated pressures strongly reduce local terrestrial biodiversity^6^. For this reason, land use change is becoming a force of global importance, although it has generally been considered a local environmental issue^7^. Several decades of research have repeatedly demonstrated the declines in biodiversity due to the loss, modification, and fragmentation of habitats, degradation of soil and water, and over exploitation of native species^8^.

Protected areas (e.g. nature reserves and natural parks) represent a cornerstone of efforts to safeguard biodiversity and provide a host of ecosystem services based on providing refuges to biodiversity and limiting human impact on biodiversity^9–11^. The global coverage of protected areas has increased rapidly from 1990 (13.4 million km^2^) to 2014 (32 million km^2^), with a total of 209,000 protected areas that cover 3.4% of the ocean and 15.4% of the terrestrial surface of the world^12^. In China, the most important protected areas are the nature reserves, which have doubled in number from 2001 (1227) to 2018 (2750), increasing the total land coverage from 9.95 to 14.83% of China^13^.

Chinese nature reserves are designed to include three functional zones, i.e. a core zone, buffer zone and experimental zone^13^. The core zone is designed to protect natural ecosystems and the important habitat of endangered species, and it is surrounded by a buffer zone to human impacts on natural ecosystems and the habitat of the species. The experimental zone, which surrounds the buffer zone, allows human development. These zoning patterns were designed to emphasizing the coordination of species and habitat diversity, while promoting harmony between human development and wildlife^14^. Although China’s nature reserves serve moderately well for birds and mammals, many other major taxa that are key regulators of ecosystems services are not well protected^2^. Assessing the efficacy of protected areas for conservation is a critical role of conservation biology^11,15–17^.

As one of the most threatened groups of birds, galliforms (Order Galliformes) are sensitive to land use change and habitat degradation^17,18^. Brown Eared-pheasant (*Crossoptilon mantchuricum*) is a globally threatened galliform endemic to forests in northern China^19^. This pheasant is currently listed as vulnerable to extinction IUCN red list of Threatened Species^20^ and classified as high priority for conservation in China^21–23^. Populations of Brown Eared-pheasant have declined over the twentieth century as their habitat became increasingly isolated and fragmented^24^. Currently, there are only three populations limited to the Luliang Mountains of western Shanxi Province, the mountains of north-western Hebei Province, and western Beijing and central Shaanxi Province^24^.

The east population of Brown Eared-pheasant has been long isolated and has the lowest population density^25^. The Xiaowutaishan National Nature Reserve (XNNR) and the Baihuashan National Nature Reserve (BNNR) were established in 2002 and 2008, respectively, to protect Brown Eared-pheasant. These two national nature reserves were also established as a part of eight sibling national nature reserves for Brown Eared-pheasant conservation. However, these reserves have had mixed success. Although the density of BNNR populations increased from 0.98 individual/km^2^ in 1993 to 2.66 individual/km^2^ in 2017^26,27^, the density of XNNR populations decreased from 11.45 individual/km^2^ in 1996 to 3.68 individual/km^2^ in 2017^26,28^.

To assess the ability of nature reserves to conservation of endangered species in highly urbanized regions, we used the Brown Eared-pheasant as a case study. Specifically, we used species distribution models (SDMs) and fragmentation analysis to assess the spatial and temporal dynamics of eastern populations of Brown Eared-pheasant. Our objectives were to: (1) assess Brown Eared-pheasant habitat suitability in the highly urbanized area of the eastern populations; (2) explore habitat change from 1995 to 2013 to identify conservation gaps; and (3) analyze the degree of habitat fragmentation. Our results provide proscriptive conservation information for the Brown Eared-pheasant and the two nature reserves, as well as demonstrate how conservation strategies can be formulated in highly urbanized regions.

## Results

### Distribution size and range shifts

**Figure 1:**
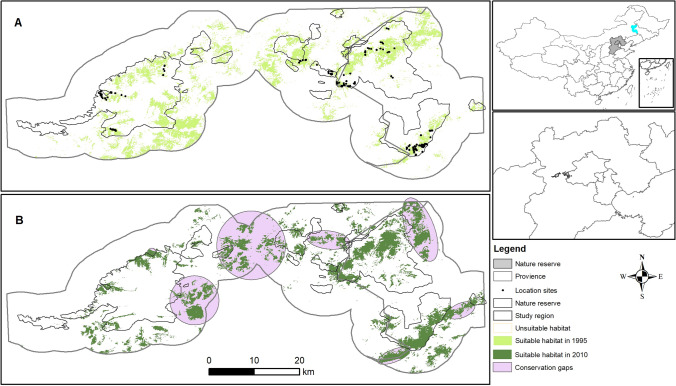
Study area and predicted suitable habitat of the eastern population of Brown Eared-pheasant. Predicted suitable habitat in (**A**) 1995 and (**B**) 2013. Gaps in conservation, where highly suitable habitat falls outside of nature reserves, are highlighted in pink.

**Figure 2:**
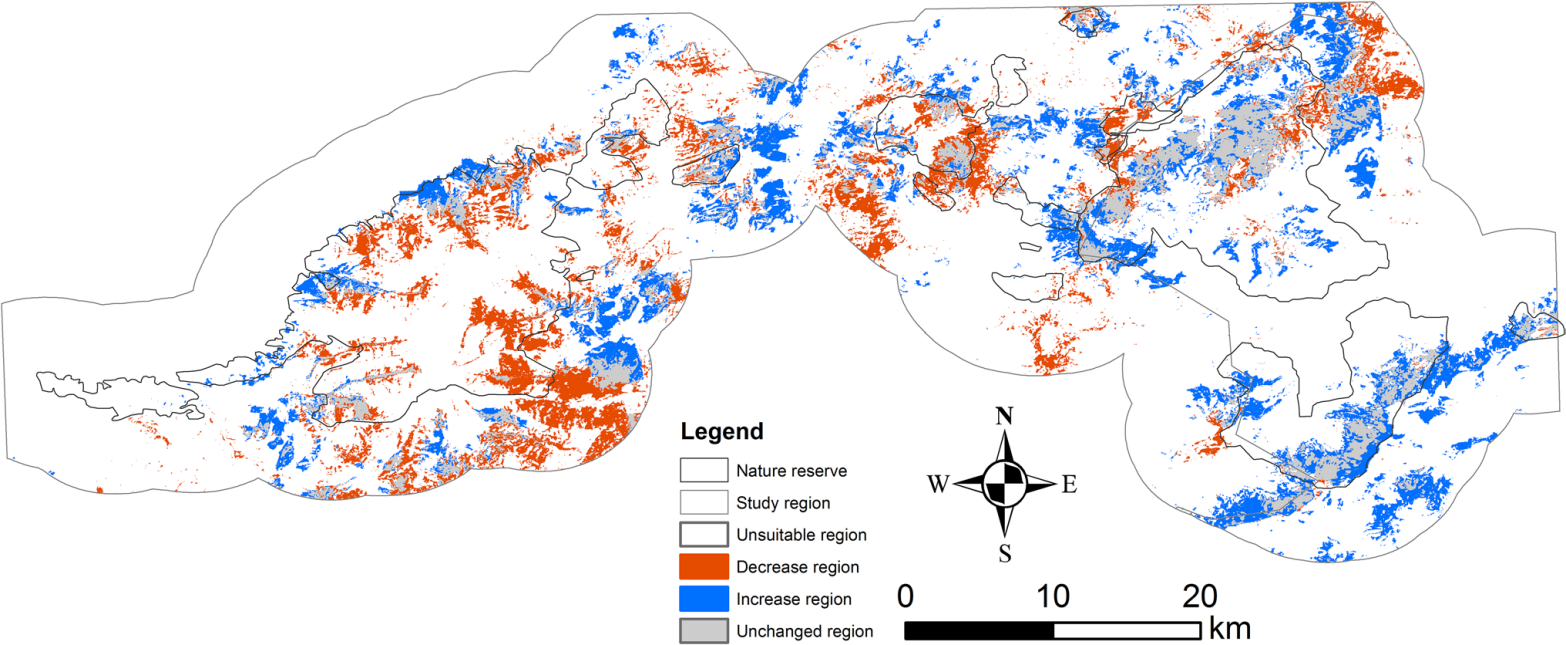
Changes in suitable habitat of Brown Eared-pheasant from 1995 to 2013. The areas without color within the study region and Nature Reserve is unsuitable region.

Our MaxEnt models performed well (AUCs 0.960 and 0.963 in 1995 and 2013, respectively) for the Brown-Eared-pheasant (Fig. 1). Our models predicted that habitat suitability in 2013 was largely concentrated in the two nature reserves, and the area of habitat suitable has remained relatively constant from 1995 (225 km^2^) to 2013 (248 km^2^) (Table 1). However, the distribution of suitable habitat has shifted largely (Fig. 2). Habitat loss and gain has resulted in a latitudinal shift in predicted suitable habitat. Between 1995 and 2013, we found that 43% of total 1995 suitable habitat remained unchanged, but 58% was lost and 68% newly gained. GAP analysis revealed that the area of suitable regions in nature reserves has remained relatively constant overall, but has increased in BNNR and declined in XNNR (Table 1, Fig. 1). The suitable region in BNNR increased from 20.3% in 1995 to 29.2% in 2013, whereas the suitable areas in XNNR declined from 25.5% in 1995 13.2% in 2013. Furthermore, more than half suitable areas expanded outside of these two nature reserves.

**Table 1:** Suitable habitat of the Brown Eared-pheasant in 1995 and 2013. NR, Suitable habitat in Nature Reserve; Gaps, Suitable habitat outside nature reserves.

Year	Area of suitable region and percent							
	Suitable	BNNR	XNNR	Gaps	NR	Decreased	Increased	Unchanged
1995	225 km^2^	46 (20%)	58 (26%)	122 (54%)	103 (46%)	0	0	225 km^2^
2013	248 km^2^	73 (29%)	33 (13%)	143 (57%)	105 (42%)	130 (58%)	152 (68%)	96 (43%)

### Habitat fragmentation

**Figure 3:**
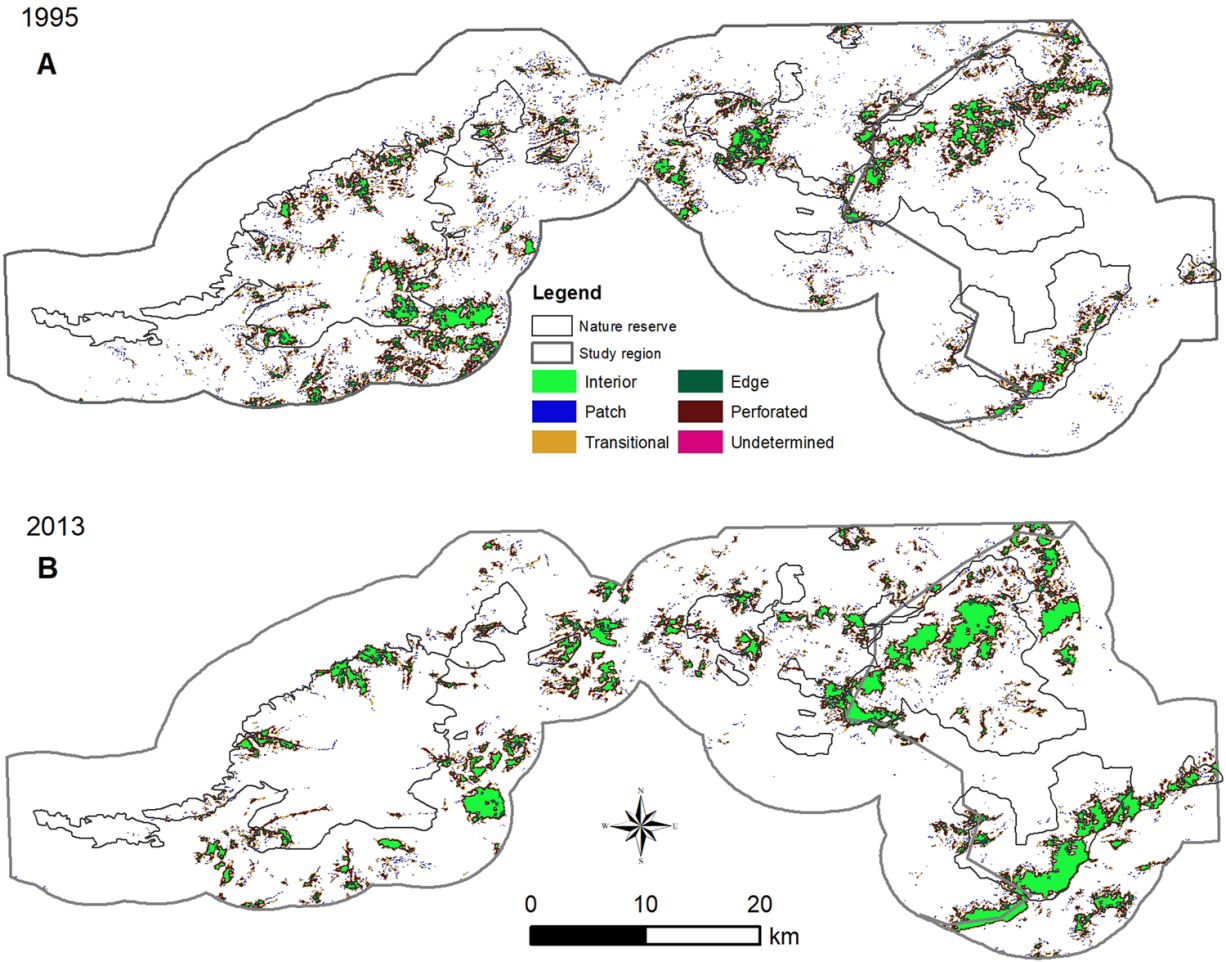
Habitat fragmentation maps of Brown Eared-pheasant in 1995 (**A**) and 2013 (**B**).

Our analysis of functional categorization of habitat fragmentation revealed that Interior habitat areas, representing a highly suitable habitat, increased from 25.26 km^2^ in 1995 to 67.17 km^2^ in 2013 (Fig. 3, Table 2). More fragmented Patch habitat areas declined from 35.07 km^2^ in 1995 to 20.93 km^2^ in 2013.

**Table 2:** Area occupied by each habitat fragmentation category.

	1995	2013
Interior	25.26 km^2^	67.17 km^2^
Patch	35.07 km^2^	20.93 km^2^
Transitional	41.46 km^2^	34.73 km^2^
Edge	32.86 km^2^	30.71 km^2^
Perforated	88.99 km^2^	93.25 km^2^
Undetermined	0.11 km^2^	0.09 km^2^

## Discussion

### Land use change threats

Our study highlights the important roles that national nature reserve can play in conservation of an endangered pheasant species in a highly urbanized area. The study region contains the largest urbanized region in Northern China, which accounts for a significant portion of Chinese GDP and is home to 110 million people^4,5^. Our results show a slight increase in suitable habitat for the Brown Eared-pheasant from 1995 to 2013, and much of the suitable habitat lies in national nature reserves. Brown Eared-pheasant populations have increased since they were discovered in 1998 in Beijing areas despite high human population density and activity^29^. However, we found that suitable habitat has shifted greatly. Although total suitable habitat has remained relatively constant, BNNR in Beijing contained 29.2% in 2013, as compared to 20.4% in 1995, while XNNR has declined from 25.5% of the total suitable habitat to just 13.2%. What is more important for conservation efforts is that more than half suitable areas expanded outside of these two nature reserves.

In general, land use change was predicted to affect pheasants through changes in spatial structure of suitable areas, and land-use and land-cover changes affect local, regional, and global climate processes^30^. The greatest threats to terrestrial biodiversity are accelerated climate change and destruction of natural habitats through direct human activities^31^. Substantial range contractions and species extinctions were caused by land use change over just the past few decades^8,32–35^. As one of the biggest urbanized regions in the world, the development of the JJJ region highly influenced land use and land cover, which directly impact local and regional species. The Brown Eared-pheasant as a vulnerable species on the IUCN Red List of Threatened Species^20^ that has a restricted range (< 13,000 km^2^) and small population size (< 17,900 birds;^23^). In our model, we used the changes in vegetation type and human interference instead of land use change. These land use changes have shifted the area of suitable habitat for the threatened eastern populations of Brown Eared-pheasant. In general, with the development of regional urbanization, land use will continue to change and suitable habitat will disappear with increasing fragmentation and pollution^36^. As a result, species’ extinction risks will grow while population sizes decline^36,37^. In this region, the total areas of suitable habitat have largely been constant, but much of it lies outside of protect areas.

### Nature reserves

We found that predicted suitable areas of Brown Eared-pheasant have different change in this two nature reserves. In totally, the suitable habitat has only slightly increased from 1995 to 2013 since the XNNR and BNNR were established in 2002 and 2008, respectively, although fragmentation has decreased. Local forest management can play an important role in the conservation of threatened species in nature reserve^38^. Our work also revealed that land use change, especially vegetation change, may be impacting the conservation of Brown Eared-pheasant in these two nature reserves. The areas of suitable habitat in XNNR declined from 1995 to 2013, which is contrary to being in BNNR (Fig. 2). Compare to BNNR, the strict management and less personal activities leading the dense forest in XNNR is the main reason of the declining in suitable habitat of Brown Eared-pheasant. Because the Brown Eared-pheasant need slight personal activities in their habitat, which is similar with the Reeves’s pheasant (*Syrmaticus reevesii*)^39^.

In the other hand, nature reserves have played a fundamental role in the conservation of species and benefit for people, which will become even more important in the future^40–42^. Our results showed that the BNNR play an important role in the conservation of the suitable habitat of Brown Eared-pheasant (Figs. 1, 2), which can be shown in the increasing of suitable habitat areas (Fig. 2, Table 1) and the decreasing of habitat fragmentation (Fig. 3, Table 2). Habitat fragmentation metrics are critical for assessing the extinction risk and conservation management strategies of threatened species^43^. Some vulnerable species in need of protection require specific management interventions to ensure its continued survival such as the Reeves’s pheasant^39^. Our study suggested that these two nature reserves may have benefitted populations of the Brown Eared-pheasant by decreasing fragmented habitat. Our results also showed that the suitable habitat areas enlarged out of the nature reserves (Figs. 1, 2). There are two main reasons which caused the results. One main reason is that the suitable habitat “forest” enlarged by the government actions which include the start of “Project of wildlife protection and Nature Reserve construction” and “Million acres of large afforestation in Beijing”. For the other thing is that Brown Eared-pheasant had a strong dispersal ability, which can help them to adapted to slight fragmented habitat^25^.

### Conservation implications

Nature reserves perform multiple roles, including conservation of particular species and biodiversity and conservation of ecosystems^44^. Establishing protected areas remains one of the most effective efforts for conserving endangered species, and more than 200,000 such areas established worldwide^10^. China has many nature reserves established for special endangered species, such as the giant panda (*Ailuropoda melanoleuca*), the tiger (*Panthera tigris amoyensis*), and Brown Eared-pheasant^44^. Assessing management effectiveness of nature reserves for endangered species is necessary for policy makers to design conservation schemes. Our study also suggested that slight frequent personal activities, and human interference in nature reserves is an effectiveness actions in the conservation of endangered species. What’s more, mapping habitat conservation redlines were successfully used with the endangered giant panda^3^ and this procedure could be applied to other endangered species such as the Brown Eared-pheasant.

The GAP analysis of nature reserves and suitable habitat of Brown Eared-pheasant showed that there are large areas of suitable habitat that fall outside of nature reserves. These results clearly show how we can expand protect lands to aid in conservation of this Brown Eared-pheasant, and potentially many other species as the climate continues to change rapidly. The boundary of nature reserves should be treat as a whole and not divided by administrative units, which is a common problem in China. Now the China government adopted the guidance on the integration of nature reserve, such as establish national forest parks, to solve above problems in China. The region JJJ is a large economic region with high human population density activity that can serve as an example for how we can protect and conserve species not only in China, but around the world.

## Methods

### Study area

The study area includes the XNNR and BNNR regions and their surrounding areas in two provinces (Beijing and Hebei, respectively) of China (Fig. 1), which contain the eastern populations of Brown Eared-pheasant^24^. These two reserves have mountainous monsoon climates in a warm temperate zone, characterized by a rainy and hot summer and a longer winter; roughly 6.4℃ annual average temperature and 700 mm annual mean precipitation^45^.

### Bird records and environmental predictors

Occurrence data for Brown Eared-pheasant was obtained from 22 line transects with a total length of 55.78 km in 2013 and 2014 in BNNR and XNNR. These resulted in 164 occurrence records include observed birds records, activity records: 112 in BNNR^46^ and 52 in XNNR (Fig. 1). Based on previous findings on the habitat preference of this pheasant, we selected 3 different data categories (habitat, topographic, and human interference) corresponding to 6 environmental variables (i.e. vegetation type, altitude, slope, aspect, distance to a residential area, and distance to a road) to construct the MaxEnt model for Brown Eared-pheasant. These six variables are generally considered to be related to species’ life history traits such as habitat utilization, foraging behavior, nest site preference and predator avoidance ^47,48^. The 30 m DEM dataset including altitude, slope, and aspect were obtained from the Geospatial Data Cloud (https://www.gscloud.cn/). The habitat variable vegetation type was obtained from the ecosystem and ecological function zoning in China database (https://www.ecosystem.csdb.cn/). We used Daogle (an open source software, https://www.daogle.com/) to acquire the basemap of China from Google Earth. We then obtained residential areas and roads in 1995 and 2013 from the basemap which was acquired from Google Earth^49^, which were used to create a Euclidean distance layer of residential areas and roads using ArcGIS 10.1. We transformed all environmental predictors into a spatial resolution of 30 m × 30 m. The band collection statistics in the Spatial Analyst extension of ArcMap 10.1 were used to calculate correlations between model variables^50^. If model variables were highly correlated (R^2^ > 0.75), we retained the variable deemed to be more ecologically relevant^51^.

### MaxEnt model construction

To model and compare the habitat suitability of Brown Eared-pheasant in 1995 and 2013, we used a maximum entropy algorithm in MaxEnt ver. 3.4.0^52^. This model has increasingly been used to model species distributions across fragmented landscapes and assess the impacts of habitat fragmentation^53^. In order to evaluate the predictive power of the MaxEnt model, we selected the area under the receiver operating characteristic curve (AUC)^54^ to evaluate the predictions using training and test data. Models were considered potentially useful for AUC values above 0.75^55^. To explore the impact of Land Use and Land Cover Change (LUCC) on the distribution of Brown Eared-pheasant, we used the habitat factors and human interference except topographic to construct models. We used the recommended default MaxEnt settings: convergence threshold (10^–5^), regularization multiplier (1), maximum number of iterations (500), and logistic output with suitability values ranging from 0 to 1^55^. We used cross-validation with five replicates to assess the robustness of SDM^50,54,56^, and the set of location records was partitioned as 80% training data and 20% testing data during each replicate. During the modeling process, we used jackknife analyses of the regularized gain with training data to examine the importance of predictors^50^.

### Suitable habitat changing and fragmentation assessments

We used a new threshold of the average predicted probability of the model-building data to transform the habitat-suitability results into presence–absence distributions^53,57,58^. We used a geographic approach for protection of biodiversity and employed GAP analysis to measure suitable habitat change^59^ in ArcGIS 10.1. Furthermore, we evaluated habitat fragmentation quantitatively and compared it between 1995 and 2013 using a fragmentation analytical model^60^. We assigned each grid of 9 × 9 pixels in which the species was present to one of six categories (Interior, Pf = 1.0; Patch, Pf < 0.4; Transitional Pf = 0.4; Perforated; Edge; and Undetermined)^51^. The amount of suitable habitat (Pf) and its occurrence in adjacent pixels (Pff) were calculated within fixed-sized windows surrounding each pixel^53,60^. We assessed the suitable habitat areas using the ʽInteriorʼ value and assessed habitat fragmentation areas using the ‘Patchʼ, ʽTransitionalʼ and ʽEdgeʼ values in this functional habitat categorization.
